# Bioinformatic analysis of FOXN3 expression and prognostic value in pancreatic cancer

**DOI:** 10.3389/fonc.2022.1008100

**Published:** 2022-10-17

**Authors:** Wei Yu, Yongkang Diao, Yi Zhang, Ying Shi, Xiangkang Lv, Chengwu Zhang, Kangjun Zhang, Weifeng Yao, Dongsheng Huang, Jungang Zhang

**Affiliations:** ^1^ Department of Postgraduates, Bengbu Medical College, Bengbu, China; ^2^ Department of Hepatobiliary and Pancreatic Surgery and Minimally Invasive Surgery, Zhejiang Provincial People’s Hospital, Affiliated People’s Hospital, Hangzhou Medical College, Hangzhou, China; ^3^ Cancer Center, General Surgery, Zhejiang Provincial People’s Hospital, Affiliated People’s Hospital, Hangzhou Medical College, Hangzhou, China; ^4^ Obstetrics and Gynecology, Zhejiang Provincial People’s Hospital, Affiliated People’s Hospital, Hangzhou Medical College, Hangzhou, China; ^5^ Key Laboratory of Gastroenterology of Zhejiang Province, Zhejiang Provincial People’s Hospital, Affiliated People’s Hospital, Hangzhou Medical College, Hangzhou, China

**Keywords:** FOXN3, pancreatic adenocarcinoma, TCGA, prognosis, immune infiltration

## Abstract

In most cancers, forkhead box N3 (FOXN3) acts as a transcriptional inhibitor to suppress tumor proliferation, but in pancreatic cancer, the opposite effect is observed. To confirm and investigate this phenomenon, FOXN3 expression in various carcinomas was determined using GEPIA2 and was found to be highly expressed in pancreatic cancer. Kaplan-Meier plotter was then used for survival analysis, revealing that high FOXN3 expression in pancreatic cancer might be associated with a poor prognosis. Similarly, clinical samples collected for immunohistochemical staining and survival analysis showed consistent results. The RNA-seq data of pancreatic cancer patients from the TCGA were then downloaded, and the differential expression gene set was obtained using R for gene set enrichment analysis (GSEA). The intersection of the above gene sets and FOXN3-related genes was defined as related differentially expressed gene sets (DEGs), and enrichment analysis was performed using Gene Ontology (GO) and the Kyoto Encyclopedia of Genes and Genomes (KEGG). Finally, we analyzed the relationship between FOXN3 and immune infiltration in pancreatic cancer. Collectively, our findings reveal that FOXN3 is involved in the occurrence and progression of pancreatic cancer and may be useful as a prognostic tool in pancreatic cancer immunotherapy.

## Introduction

Pancreatic cancer (PC) is one of the most deadly malignant tumors, with a five years relative survival rate of only 9% due to the unsatisfactory surgical outcome, difficulties of early diagnosis and high metastasis rate (1). PC rank the third leading cause of cancer-related death, and its incidence has rised over the past years (2). Pancreatic ductal adenocarcinoma (PDAC) accounts for about 85% of all PCs (3). It is, therefore, the most common histological pancreatic cancer subtype representative of PC (4). As a result, understanding the FOXN3-related molecular mechanisms of PDAC is critical for early diagnosis and targeted treatment strategies.

The proteins encoded by the Forkhead box (FOX) gene family are involved in the expression of important genes that regulate the development, proliferation and aging of various organs (5). For instance, the FOXO subfamily is involved in regulating metabolism, antioxidant stress, and cell cycle arrest (6, 7). In contrast, the FOXP subfamily is primarily involved in immune response, with FOXP3 being a key marker of Treg cells and is critical for immune homeostasis and self-tolerance (8). Meanwhile, FOXA has been shown to promote the binding of nuclear hormone receptors (9). As a result, the FOX family has received a lot of attention and is considered a potential therapeutic target in many diseases.

**Figure 1:**
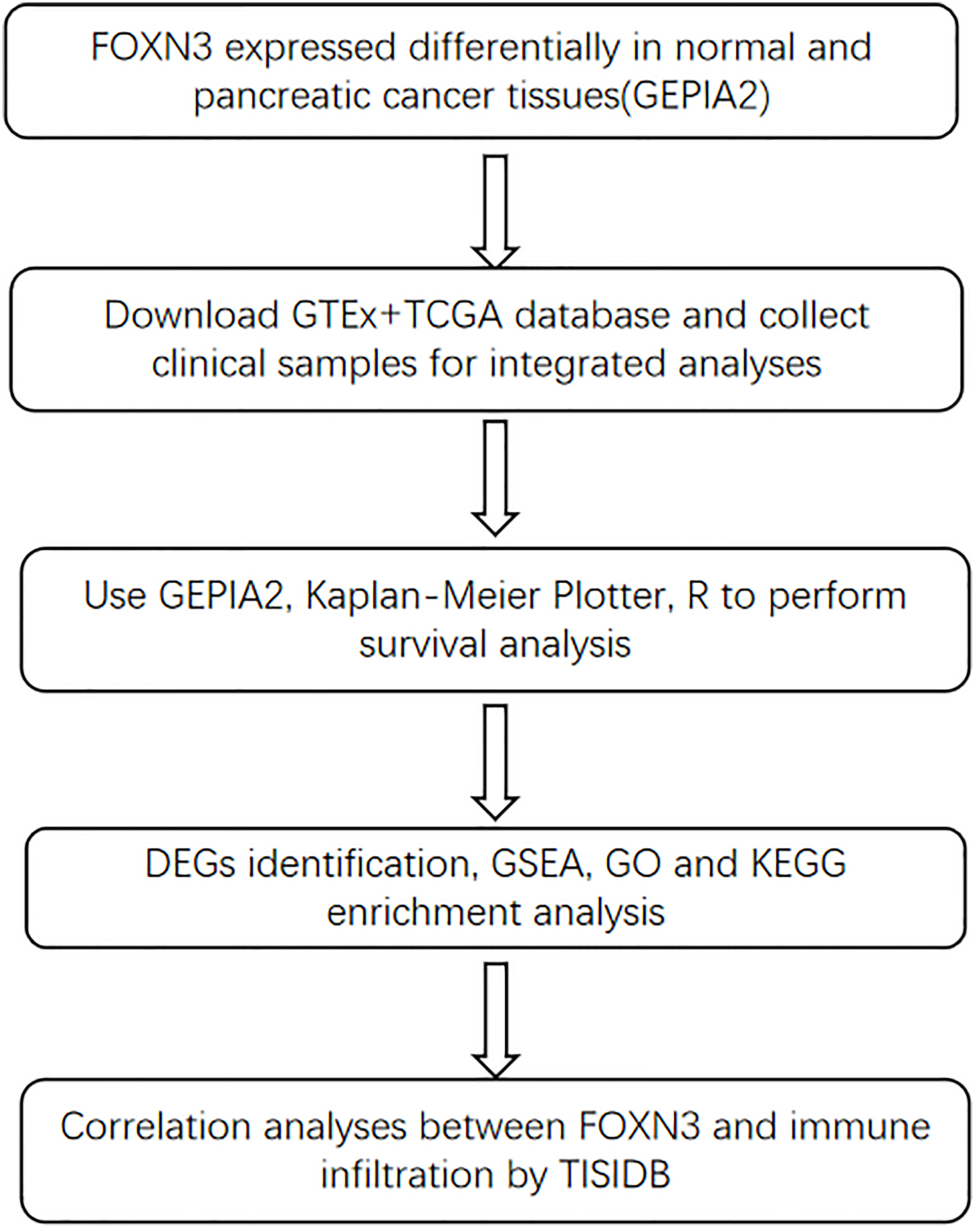
The workflow flow chart.

Pati et al. first isolated FOXN3 from yeast cells and identified multiple checkpoint mutations (10). Since FOXN3 has the ability to suppress many DNA damage-activated checkpoint mutations, it is also known as checkpoint suppressor 1 (CHSE1). FOXN3 is a member of the forkhead box family, which has a similar DNA-binding domain and is involved in organ differentiation, development, cell growth, and cancer (11). FOXN is divided into six subclasses (FOXN1–FOXN6), with FOXN3 being the only member lacking transcriptional activation domains (12). Previous studies revealed that FOXN3 could regulate other genes as a transcription factor in a variety of cancers. Moreover, the FOXN3-NEAT1-SIN3A complex was found to promote breast cancer cell diffusion and metastasis *in vivo (* 13). FOXN3 possesses a unique forkhead box region that can interact with the promoter of the E2F5 transcription factor, inhibiting hepatocellular cancer cell proliferation by downregulating E2F5 mRNA and protein expression (14). The downregulation of FOXN3 could inactive the Wnt/β-catenin pathway to promote the growth and invasion of papillary thyroid cancer and colon cancer (15, 16). Nevertheless, the clinical significance of FOXN3 expression in PC remains indistinct. The present study aims to assess the clinicopathological value and prognostic significance of FOXN3 expression in pancreatic cancer. The study workflow is shown in **Figure 1**.

## Materials and methods

### GEPIA2 database analysis

GEPIA2, a web server for analyzing the RNA sequencing expression data from The Cancer Genome Atlas (TCGA) and the Genotype-Tissue Expression project (GTEx) (http://gepia2.cancer-pku.cn), was used to analyze the expression and prognosis of FOXN3 in PDAC (17).

### Screening of differentially expressed genes (DEGs)

The RNA-seq data of TCGA_PDAC (source: https://xenabrowser.net/datapages/ n=177) was used to identify DEGs between FOXN3 high and low expression subgroups; defined by the median expression level of FOXN3. Using the “DESeq2” R package screened the DEGs, and the heatmap was constructed using the “pheatmap” R package. P <0.05, log FC > 1.5 was used to define significantly up-regulated DEGs, while P<0.05, log FC <-1.5 was used to identify significantly down-regulated DEGs.

### Kaplan–Meier plotter

Kaplan-Meier plotter was utilized to estimate the effect of genes on survival in different cancer types (http://kmplot.com) (18). The PDAC samples from TCGA database were divided into two groups according to the cutoff points determined for FOXN3 expression level. Overall survival (OS) was defined as the time from initial diagnosis of PDAC to the time to death or the last follow-up, while recurrence-free survival (RFS) was defined as the time from the first diagnosis to disease relapse. The hazard ratio (HR) and p-value had been labeled.

### Enrichment analysis

Gene ontology (GO) analysis consists of three parts, biological processes (BP), cellular component (CC), and molecular function (MF), that explore the biological function of the gene of interest from different aspects. In contrast, Kyoto encyclopedia of genes and genomes (KEGG) pathway analysis is used to further identify enriched biological pathways. Here, we used R to perform GO and KEGG pathway analysis based on DEGs with low expression vs. high expression of FOXN3 and FOXN3-related genes.

### TISIDB

The Tumor and Immune System Interaction Data Base (TISIDB) (http://cis.hku.hk/TISIDB/index.php) is an online platform that can be used to explore the relationship between the tumor and the immune system (19). The interaction between tumor cells and the immune system plays an important role in cancer initiation, progression, and treatment, so elucidating the interaction between tumor and immune system will help predict immunotherapy response and develop new immunotherapy targets. In this study, the TISIDB was used to investigate the association of FOXN3 with 28 immune cells.

### Patients and specimens

A total of 36 PDAC patients were enrolled from the Zhejiang Province People’s Hospital, China, for tumor and adjacent nontumor tissue collection. All patients were treated for the first time without any antitumor treatment such as chemotherapy and radiotherapy, and were pathologically diagnosed as PDAC after surgery. This research was approved by the Ethics Committee of Zhejiang Province People’s Hospital, and each patient agreed to a written informed consent form (QT2022276). All specimens were paraffin-embedded and fixed.

### Immunohistochemical determination

Immunohistochemical (IHC) staining of FOXN3 was conducted on paired tumors and adjacent normal tissues for 36 PDAC cases. IHC analysis was performed to investigate altered protein expression using FOXN3 antibodies (Abcam, 1:100) in 36 paraffin-embedded and archived pancreatic cancers. Two blinded independent pathologists reviewed and graded the immunostaining degree of formalin-fixed and paraffin-embedded sections. The fraction was determined by combining the proportion of positive staining tumor cells with the staining intensity. The scores from the two independent pathologists were combined into an average score for further comparative evaluation. The tumor cell proportion score was as follows: 0, no positive tumor cells; 1, <10% positive tumor cells; 2. 10%~35% positive tumor cells; 3. 35-75% positive tumor cells; 4, >75% positive tumor cells. Dyeing intensity was graded according to the following standards: 1. No dyeing; 2. Weak dyeing (light yellow); 3. Moderate staining (tan); 4. Strong staining (brown). The staining index (SI) was calculated as the product of the staining intensity score and the proportion of positive tumor cells. Using this assessment method, we assessed protein expression in malignant lesions by determining SI, with possible scores of 0, 2, 3, 4, 6, 8, 9, 12, and 16. Then the median of SI of 6 was selected as the cutoff value, and the samples with SI >6 were determined to be highly expressed, while SI ≤ 6 samples were determined to be of low expression.

### Quantitative RT-PCR

The expression of FOXN3 in pancreatic cancer and its adjacent tissues was detected by RT-PCR. Briefly, Total RNA(1 ug) was extracted(TRIzol reagent; Invitrogen, Carlsbad, CA, USA) for reverse transcription into cDNA according to the manufacturer’s instructions. Then, to get the expression level of FOXN3 mRNA, we used real-time PCR by the SYBR Green quantitative PCR kit (Invitrogen) and an Applied Biosystems 7300 real-time PCR system (Carlsbad, CA, USA) according to the manufacturer’s instructions. FOXN3 forward primer, 5′-TCTGACATGCCCTACGATGC-3′, reverse primer, 5′-CTATGCACCACAACGACCCT-3′. All data were normalized to the GAPDH (forward primer, 5’-GACAACTTTGGCATCGT-3’; reverse primer, 5’-ATGCAGGGATGATGTTC TGG-3’). Expression level of FOXN3 were calculated by a comparative Ct method.

## Results

### Distribution of FOXN3 expression in cancer tissues

**Figure 2:**
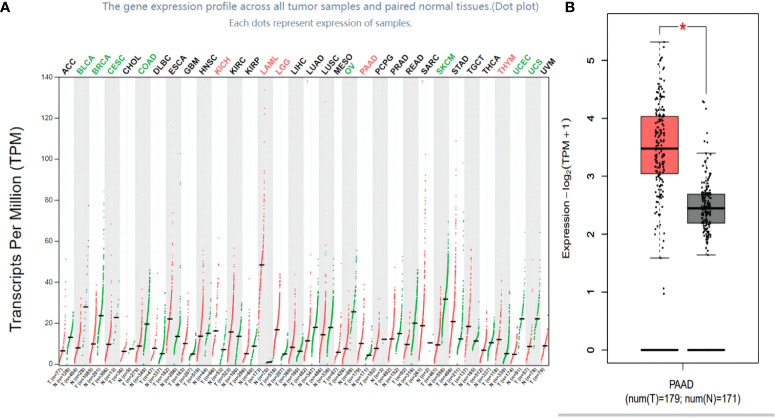
FOXN3 expression in carcinoma tissues. **(A)** FOXN3 expression in pan cancer in TCGA database. **(B)** FOXN3 expression in Pancreatic adenocarcinoma(PAAD) in TCGA database. *P < 0.05.

**Figure 3:**
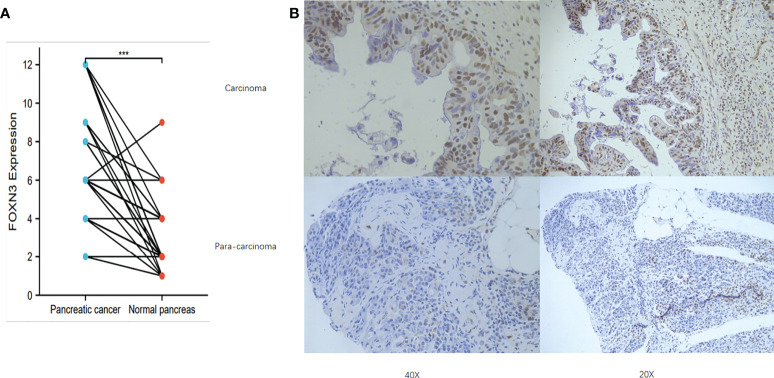
**(A)** FOXN3 expression in PC tissues and adjust tissues in clinical samples. ***P < 0.005. **(B)** Immunohistochemistry of FOXN3 expression in PC tissues and adjust tissues.

The TCGA and the GTEx database were used to analyze FOXN3 expression in generalized carcinoma on the GEPIA2 online platform, revealing that FOXN3 was down-regulated in Bladder Urothelial Carcinoma (BLCA), Breast invasive carcinoma (BRCA), Cervical squamous cell carcinoma and endocervical adenocarcinoma (CESC), Colon adenocarcinoma (COAD), etc. In contrast, FOXN3 was up-regulated in Kidney Chromophobe (KICH), Pancreatic adenocarcinoma (PDAC) and Thymoma (YHYM) (**Figures 2A, B**). Consistently, our IHC results also confirmed that FOXN3 was highly expressed in pancreatic cancer tissue samples (**Figure 3A**). The results of IHC staining showed that nucleus staining was dominant(**Figure 3B**). And we used qRT-PCR to understand the mRNA expression level of FOXN3, and the results showed that FOXN3 expression was significantly increased in cancer tissues compared with normal pancreatic tissues (**Figure S1**).

### Relationship between FOXN3 expression and survival in patients with PDAC

**Figure 4:**
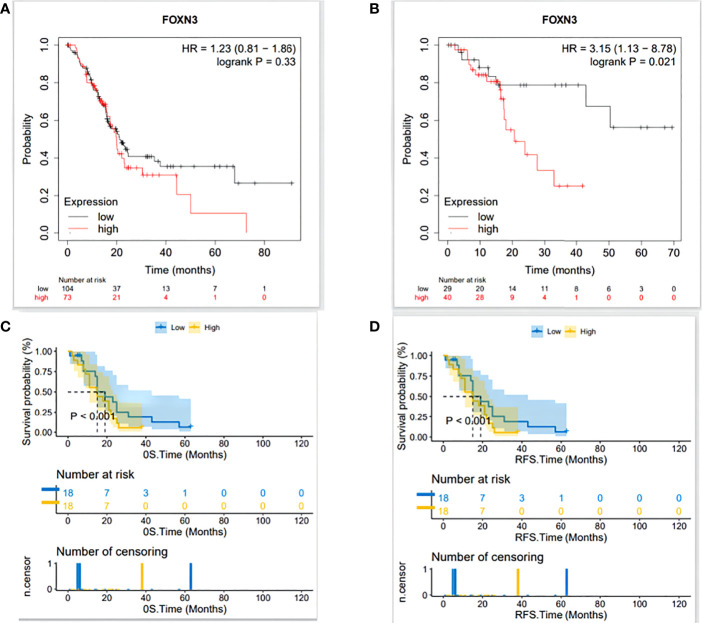
**(A)** The relationship between FOXN3 expression level and overall survival in TCGA database analyzed by Kaplan-Meier Plotter. **(B)** The relationship between FOXN3 expression level and disease-free survival in TCGA database by Kaplan-Meier Plotter. **(C)** The relationship between FOXN3 expression level and overall survival in clinical samples. **(D)** The relationship between FOXN3 expression level and disease-free survival in clinical samples.

Based on the optimal cut-off points provided by Kaplan-Meier Plotter, we divided PDAC patients into high expression and low expression groups for survival analysis. The results showed that high FOXN3 expression level was associated with poor recurrence free survival (p<0.05) but has no statistical significance for overall survival(p>0.05)(**Figures 4A, B**). In the clinical samples, Patients were divided into high expression(n=18) and low expression(n=18) groups using the median SI. Prognostic analysis revealed that patients with high FOXN3 expression had a worse disease-free survival and overall survival compared to those with low FOXN3 expression(p<0.05)(**Figures 4C, D**).

### GO, KEGG and GSEA analysis of DEGs

**Figure 5:**
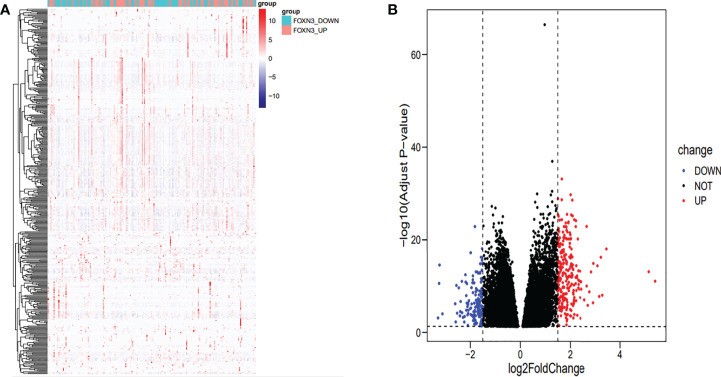
**(A)** Heat map of DEGs when FOXN3 is down-regulated and up-regulated in PDAC based on TCGA database. **(B)** Volcanic map of differential gene expression.

**Figure 6:**
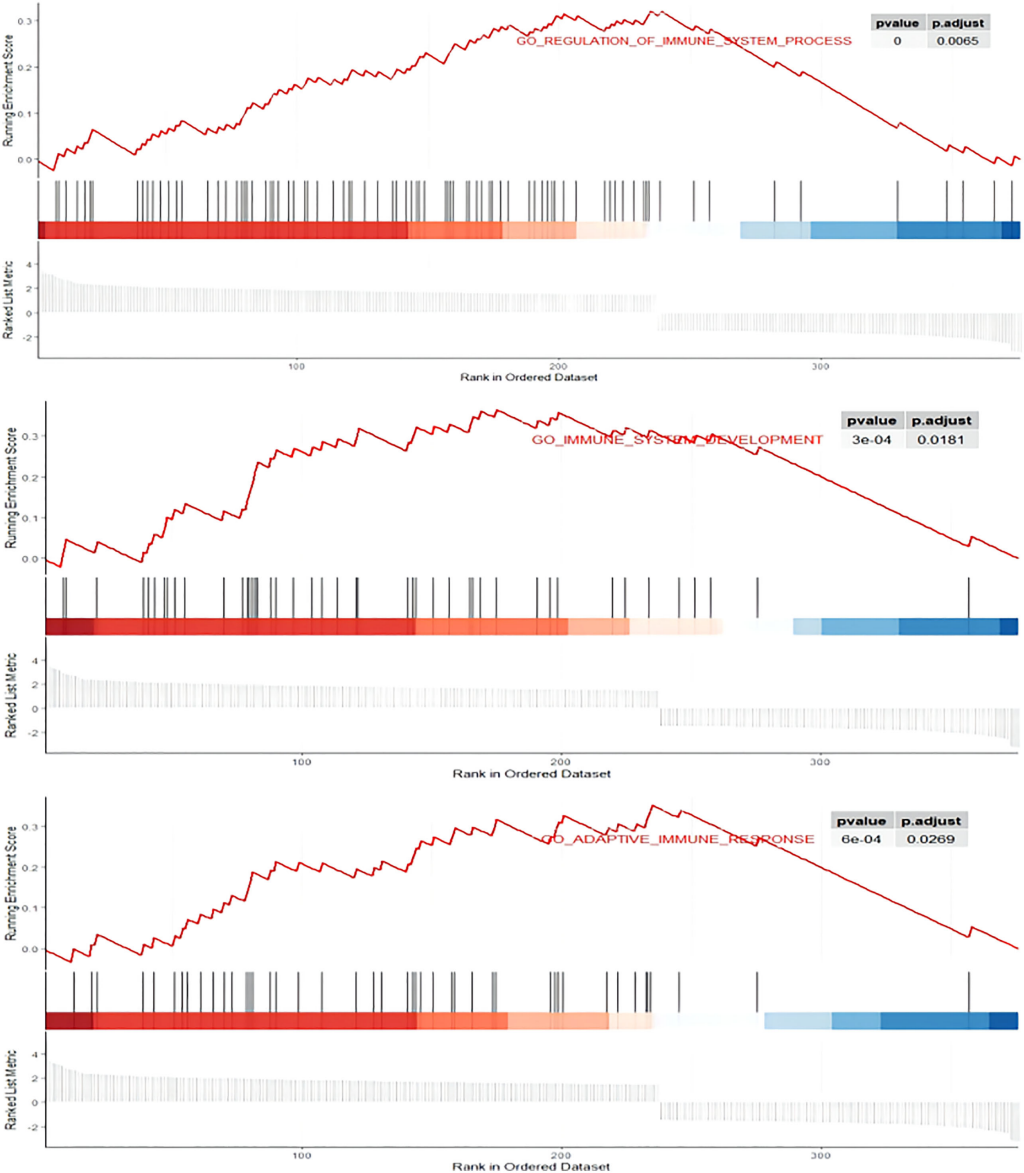
GSEA enrichment plots. DEGs was significantly correlated with the regulation of immune system process, adaptive immunity, and immune system development. P.adjust<0.05.

**Figure 7:**
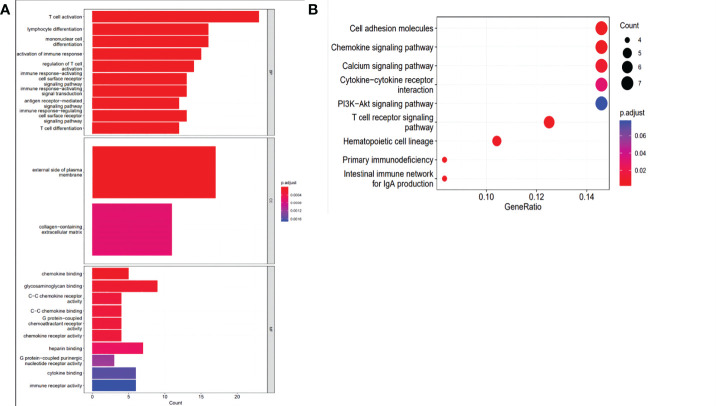
**(A)** GO analysis of the intersection of DEGs with related genes in PC biological process, cell components, and molecular function enrichment analyses. **(B)** KEGG pathway analysis of the intersection of DEGs with related genes.

A total of 394 genes (|log FC| > 1.5) from the TCGA were screened using differential expression gene analysis in pancreatic cancer patients. Next, the related heat map and volcano map were constructed accordingly (**Figure 5**). The DEGs were used for gene set enrichment analysis (GSEA), revealing that they were significantly correlated with the regulation of the immune system process, adaptive immunity, and immune system development (P < 0.05) (**Figure 6**). Based on the TCGA database, the expression correlation between FOXN3 and all genes in PDAC was analyzed, and the genes with Pearson coefficient > 0.5 were defined as FOXN3-related genes, with a total of 98 identified genes. These FOXN3-related genes were then subjected to GO and KEGG analysis, demonstrating that these genes were significantly associated with BP, especially in the T cell activation, lymphocyte differentiation, mononuclear cell differentiation, activation of immune response, regulation of T cell activation, immune response-activating cell surface receptor signaling pathway, immune response-activating signal transduction, antigen receptor-mediated signaling pathway, immune response-regulating cell surface receptor signaling pathway and T cell differentiation, to name a few. Together, these results imply that FOXN3 is closely related to the occurrence and development of the tumor immune microenvironment in PDAC tissues. Based on KEGG’s analysis, FOXN3 was found to be associated with cell adhesion molecules, T cell receptor signaling pathway, Primary immunodeficiency, Chemokine signaling pathway, etc. (**Figure 7**). Most of which affect the immune process and are in line with the above conclusion.

### The relationship between FOXN3 gene expression and immune infiltration in PDAC

**Figure 8:**
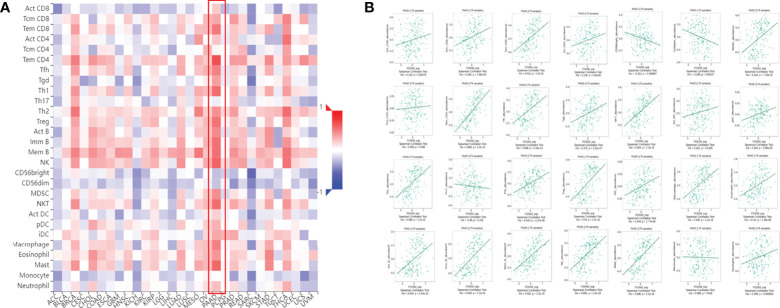
**(A)** The relationship between FOXN3 gene expression and TIICs in pan-cancer. **(B)** Relationship between FOXN3 expression and tumor-infiltrating immune cells in PDAC.

**Figure 9:**
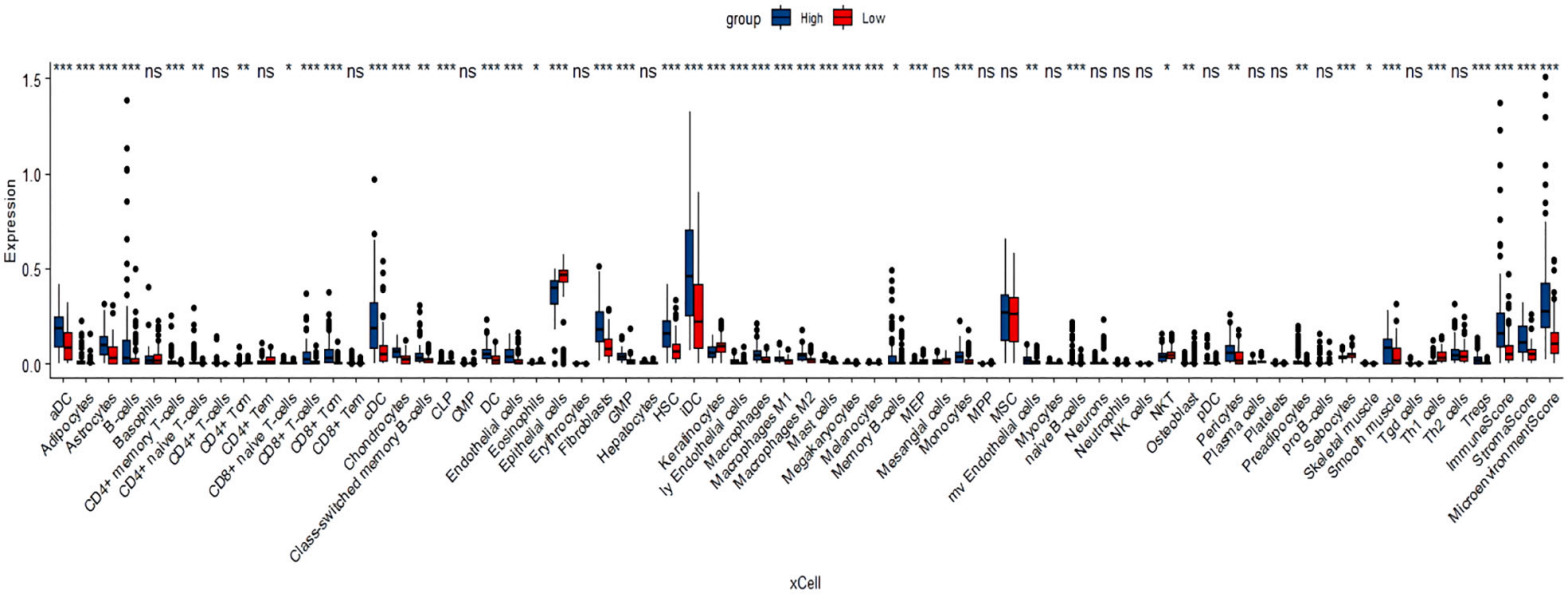
Xcell analyzed the relationship between FOXN3 and immune cell expression * means P < 0.05, ** means P < 0.005, *** means P < 0.005, “ns” means no significance.

**Figure 10:**
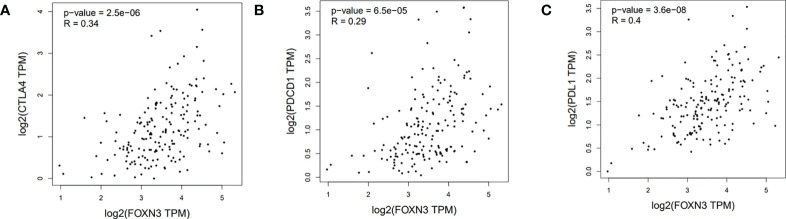
**(A)** Correlation between FOXN3 and CTLA4 in PDAC patients in TCGA database. **(B)** Correlation between FOXN3 and PDCD1 in PDAC patients in TCGA database. **(C)** Correlation between FOXN3 and PDL1 in PDAC patients in TCGA database.

Tumor-infiltrating immune cells (TIICs) include T cells, macrophages, NK cells, etc., which are important components of the tumor immune microenvironment (TIME). TISIDB was used to analyze the relationship between FOXN3 expression and TIICs in pan-cancer samples (**Figure 8A**). FOXN3 expression was found to be highly correlated with most TIICs (such as CD8T cells, CD4T cells, etc.) in PDAC. Therefore, FOXN3 may play an important role in the tumor immune microenvironment of PDAC (**Figure 8B**). To further understand how FOXN3 affects the occurrence and development of the PDAC immune process, Xcell was used to understand the relationship between FOXN3 and TIICs. Xcell is a ssGSEA-based method that calculates the abundance fractions of 64 immune cells. Herein, TCGA samples was classified into high and low groups using the median FOXN3 expression. The figure shows the differential expression of immune cells between the two groups. FOXN3 high expression group had higher immune cell expression (**Figure 9**). Moreover, using GEPIA2, we analyzed the correlation between main immunotherapy drug targets and FOXN3, including PDCD1, CTLA4 and PDL1, with a p-value less than 0.05 (**Figure 10**).

## Discussion

Since it is difficult to detect PDAC in the early stage, most PDAC patients are in advanced stages upon diagnosis, and systemic chemotherapy is their only treatment option (20). However, chemotherapy is frequently ineffective due to PDAC’s strong immunosuppressive environment (21). As a result, we urgently need to understand the immune microenvironment of PDAC to devise new treatment strategies.

During pancreatic cancer development, pancreatic stellate cells become activated and undergo fibrosis around the tumor, forming a dense stroma devoid of blood vessels that promotes immune escape by blocking lymphocyte invasion (22, 23). Therefore, pancreatic cancer is commonly infiltrated with immune cells, but the core of the tumor is relatively free of cytotoxic T lymphocytes (24), preventing adaptive immunity from accurately recognizing pathogens or tumor tissue to mount an effective response (25). Analysis of large PDAC genomic datasets showed that only a small number of PDAC were immunologically active (26). In PDAC, immunosuppressive cells, tumor-associated macrophages, myeloid suppressor cells, and Treg cells are initially prominent, forming an immunosuppressive environment (27).

FOXN3 is a member of the FOX protein family, which has been studied in the liver, thyroid, breast, and other cancers. Herein, we mainly focus on its relationship with PDAC. Based on an online database of gene differential expression, survival analysis and a combination of immunohistochemical staining results, we concluded that FOXN3 is highly expressed in PDAC and could potentially be used as a prognostic tool. To further understand the mechanism of FOXN3 in PDAC, GSEA analysis was performed using DEG, and pathway analysis revealed an enrichment in immune-related functions. Subsequently, immune infiltration analysis was performed on TISBD, and FOXN3 was found to be significantly associated with TIICs in PDAC when compared to other cancers. The expression of most TIICs increases with FOXN3 expression, implying that FOXN3 may influence the immune microenvironment in PDAC by regulating TIICs expression. Next, we searched for FOXN3-related genes and looked for intersecting DEGs. Subsequently, the final gene set was used for further GO and KEGG analysis, and the analysis results validated our above conjecture. We discovered that FOXN3’s functional role is closely related to the T cell receptor pathway, and T cells play an important role in the immunosuppressive microenvironment of PDAC.

PDAC is typically infiltrated by T cells with a low proportion of CD8T toxic cells and is often referred to immunologically as a “cold” tumor (21). Treg cells have a role in maintaining immune homeostasis in normal organisms by inhibiting inflammatory responses (28). FOXP3 positive Treg cells were observed to increase in number as PDAC progressed, leading to immune evasion (29). Taken together, an increase in the total T cell count in PDAC could contribute to tumor development and a worse prognosis (30).

Combined with our findings, it can be determined that FOXN3 plays a non-negligible role in the formation of the immunosuppressive microenvironment of PDAC by regulating T cells. In most cancers, T cells, the main force of the anti-tumor army in the immune system, are disabled by the specific immune cold environment of PDAC (31). However, if T cell immunity can be sufficiently induced, it will inhibit tumor development (32). Immune checkpoint blockers, including antibodies against cytotoxic T lymphocyte antigen-4(CTLA4), programmed cell death protein 1(PD-1) and its ligand PD-L1, enhance T cell anti-tumor immune function (33). After analyzing its relationship with FOXN3, we identified a statistically significant correlation, implying that FOXN3 could be a potential target for immunotherapy in PDAC.

Herein, the results of clinical samples and the TCGA and GTEx databases were used to validate each other in this study, but the total sample size was small, which may have resulted in significant bias. Further limitations include the lack of additional *in vitro* and *in vivo* evidence. Therefore, we look forward to future research to confirm the function of FOXN3 in PDAC.

## Data availability statement

The original contributions presented in the study are included in the article/**Supplementary Material**. Further inquiries can be directed to the corresponding authors.

## Ethics statement

The studies involving human participants were reviewed and approved by the ethics committee of Zhejiang Province People’s Hospital. The patients/participants provided their written informed consent to participate in this study.

## Author contributions

JZ, DH and YS designed and conceptualized studies. WY and YD conducted experiments, process experimental data, and conduct bioinformatics analysis. WY, YD, KZ, XL, YZ, YS, WFY, and CZ participated in editing manuscripts and put forward valuable suggestions. All authors contributed to the article and approved the submitted version.

## Funding

This work was supported by the Science and Technology Department Public Welfare Project of Zhejiang Province (GF22H168107), Zhejiang Provincial Science Foundation Committee of China (LQ19H160015) and the Medical and Health Science and Technology Project of Zhejiang Province (2022RC107, 2021KY486).

## Conflict of interest

The authors declare that the research was conducted in the absence of any commercial or financial relationships that could be construed as a potential conflict of interest.

## Publisher’s note

All claims expressed in this article are solely those of the authors and do not necessarily represent those of their affiliated organizations, or those of the publisher, the editors and the reviewers. Any product that may be evaluated in this article, or claim that may be made by its manufacturer, is not guaranteed or endorsed by the publisher.

## Supplementary material

Supplementary Figure 1 Differences in FOXN3 mRNA expression between pancreatic cancer and normal pancreatic tissues.
