# Supplementary figures and images for: Bioinformatic analysis of FOXN3 expression and prognostic value in pancreatic cancer
Source: Front Oncol. 2022 Oct 17;12:1008100. doi: 10.3389/fonc.2022.1008100 (PMC9619050; doi:10.3389/fonc.2022.1008100)

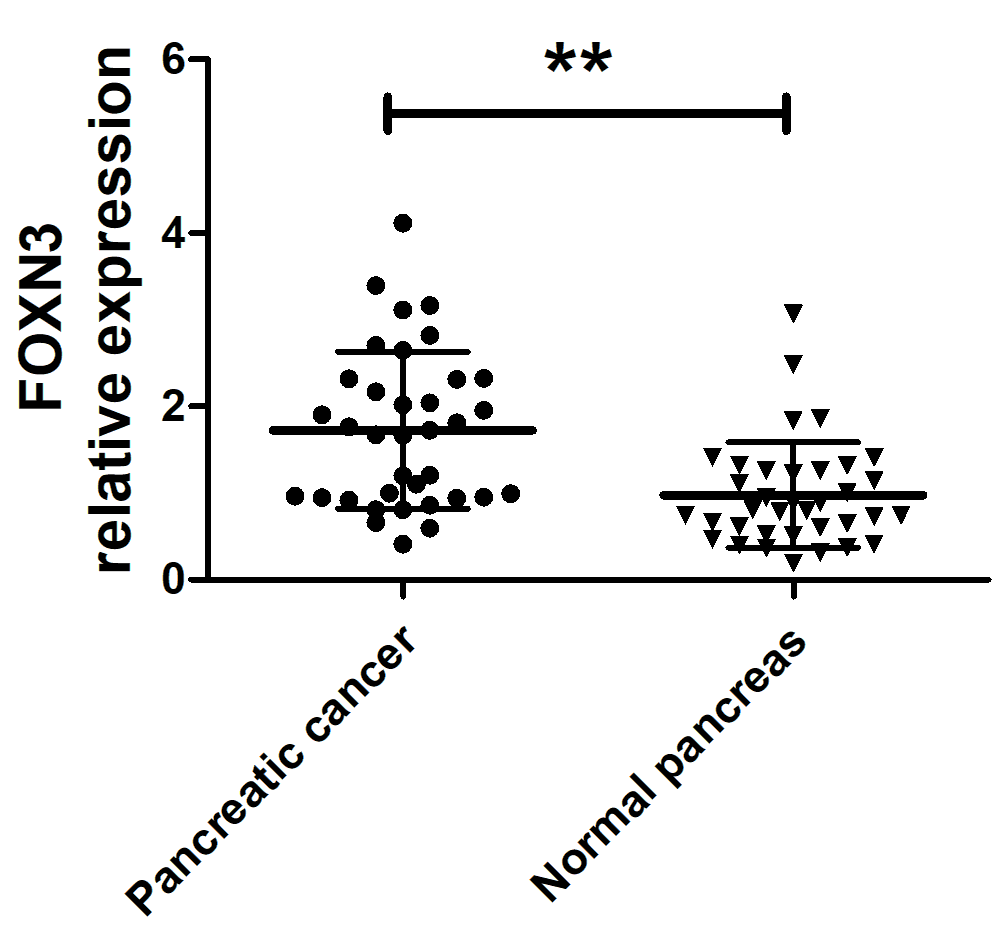

Supplement: Supplementary Figure 1 — Differences in FOXN3 mRNA expression between pancreatic cancer and normal pancreatic tissues. [file Image_1.tif]
